# Translation, validity and reliability of the British Sign Language (BSL) version of the EQ-5D-5L

**DOI:** 10.1007/s11136-016-1235-4

**Published:** 2016-02-18

**Authors:** Katherine D. Rogers, Mark Pilling, Linda Davies, Rachel Belk, Catherine Nassimi-Green, Alys Young

**Affiliations:** Social Research with Deaf People (SORD), School of Nursing, Midwifery and Social Work, University of Manchester, Jean McFarlane Building, Oxford Road, Manchester, M13 9PL UK; School of Nursing, Midwifery and Social Work, University of Manchester, Jean McFarlane Building, Oxford Road, Manchester, M13 9PL UK; Institute of Population Health, University of Manchester, Jean McFarlane Building, Oxford Road, Manchester, M13 9PL UK

**Keywords:** EQ-5D-5L, Psychometric properties, Deaf population, British Sign Language, Translation

## Abstract

**Purpose:**

To translate the health questionnaire EuroQol EQ-5D-5L into British Sign Language (BSL), to test its reliability with the signing Deaf population of BSL users in the UK and to validate its psychometric properties.

**Methods:**

The EQ-5D-5L BSL was developed following the international standard for translation required by EuroQol, with additional agreed features appropriate to a visual language. Data collection used an online platform to view the signed (BSL) version of the tests. The psychometric testing included content validity, assessed by interviewing a small sample of Deaf people. Reliability was tested by internal consistency of the items and test–retest, and convergent validity was assessed by determining how well EQ-5D-5L BSL correlates with CORE-10 BSL and CORE-6D BSL.

**Results:**

The psychometric properties of the EQ-5D-5L BSL are good, indicating that it can be used to measure health status in the Deaf signing population in the UK. Convergent validity between EQ-5D-5L BSL and CORE-10 BSL and CORE-6D BSL is consistent, demonstrating that the BSL version of EQ-5D-5L is a good measure of the health status of an individual. The test–retest reliability of EQ-5D-5L BSL, for each dimension of health, was shown to have Cohen’s kappa values of 0.47–0.61; these were in the range of moderate to good and were therefore acceptable.

**Conclusions:**

This is the first time EQ-5D-5L has been translated into a signed language for use with Deaf people and is a significant step forward towards conducting studies of health status and cost-effectiveness in this population.

## Background

Interest in the health status of populations, in particular cultural groups such as the Deaf population, continues to rise. The Deaf population (with a capital ‘D’) concerns Deaf people who use sign language and identify themselves as part of the Deaf community. Sign languages are not universal, and, in the UK, the language used by Deaf people is British Sign Language (BSL). Previous research on the health of Deaf populations mostly focused on mental well-being [1–3], although there has also been a recent growth in the literature about deficits in the physical health of Deaf people. Studies demonstrate that Deaf people have poorer mental and physical health than the majority population of hearing people and that they experience inequalities in accessing healthcare services [4]. Communication difficulties between healthcare providers and patients, patients’ lack of access to health care in their preferred language and poor health-related information in signed languages are the main factors [5, 6].

The health status of people within the general population can be measured using a standardised assessment tool developed by EuroQol: the EQ-5D (http://www.euroqol.org/eq-5d-products.html) and subsequently the EQ-5D-5L, the latter being considered more robust because it produces fewer ceiling and floor effects [7]. The EQ-5D-5L is a self-report tool which includes five dimensions of health: Mobility, Self-Care, Usual Activities, Pain/Discomfort and Anxiety/Depression, and a Visual Analogue Scale (VAS) designed to give an overall, self-report summary evaluation of an individual’s health status.

Although population norms are available for the EQ-5D for the UK in its English (three level) version [8], the Deaf population in the UK use BSL as their first or preferred language and constitute a separate cultural community [9, 10]. Theoretically, English in its written form would seem to present no barriers to access because it is not dependent on hearing, but it is not an appropriate format for a population whose main language is other than English [11]. The socio-economic, educational and cultural experiences of Deaf people are also different from mainstream society [12, 13]; therefore, the value of scores developed for the general population in the UK is questionable. There is currently no version of the EQ-5D in BSL, or in any other signed language.

The EQ-5D can be used to estimate health benefits in terms of quality-adjusted life years (QALYs) for use in economic evaluations to assess the relative cost-effectiveness of healthcare interventions [14]. The QALY is the measure of health benefit preferred by National Institute for Health and Care Excellence (NICE) for such analyses [14]. Where the population’s set of health preferences are not known, then ‘one from a nearby or “similar” population’ [15] can be used. Deaf populations are not similar to those with hearing loss because the latter group will not use a signed language and are not members of the cultural community denoted by BSL. This means that further investigation is required to identify (or, if necessary, develop) a generic health status measure that is relevant and culturally appropriate for the Deaf population.

The study aims were to: (1) translate the English version of EQ-5D-5L into BSL; (2) validate the EQ-5D-5L BSL on a Deaf population of BSL users in the UK; (3) investigate the psychometric properties of the EQ-5D-5L BSL to establish its reliability. This provides a basis for further research to validate existing norms for the health domains and preference (utility) weights attached to the EQ-5D which were developed for the general population. This work, however, was outside the scope of this project.

## Methods

### The translation

Work on the translation and reliability testing of various standard assessments into BSL (including the CORE-OM, PHQ-9 and GAD-7) has previously been carried out by the authors and specific challenges resulting from translating from a written form into a visual form of a language discussed [13, 16, 17]. The resulting robust translation protocols arising from previous work were applied to the translation of the EQ-5D-5L. Two translation teams were established. The forward translation team consisted of two native Deaf BSL users who are experienced translators, fluent in written English; the back translation team consisted of two registered interpreters (one Deaf and one hearing) who are bilingual in BSL and English. Both teams translated from their second language into their first. The work was overseen by a native BSL user (the first author) who is bilingual in BSL and English. The EuroQol group translation guidelines [18] were adhered to, but adapted to take into account the fact that BSL is a visual (non-written) language. This meant that each stage of the translation procedure was filmed and recorded to allow comparisons between versions. Team discussions concerning discrepancies between forward and back translations were also carried out in BSL and filmed so that points could be referred back to when considering amendments. Team discussions resulted in consensus on the translation of each item to be used in the subsequent draft having considered reasons underlying any differences between the forward- and back-translated versions.

#### Forward translation

**Fig. 1:**
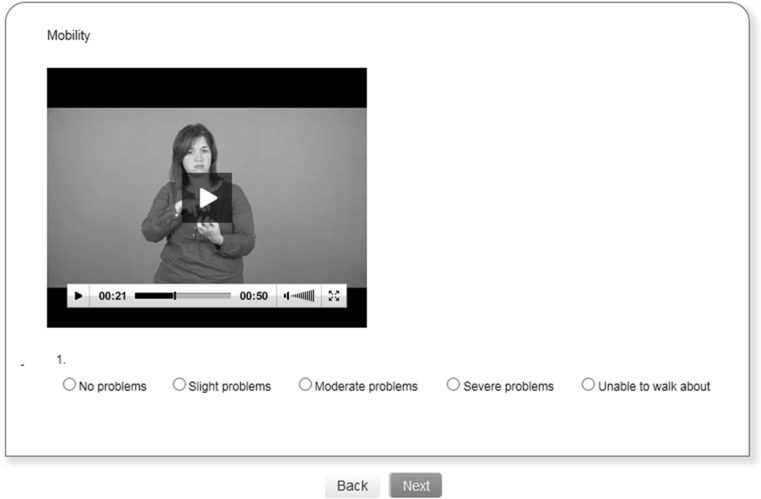
Example of the on-screen EQ-5D-5L in BSL

 The two forward translators independently translated the EQ-5D-5L into BSL (first draft). A key problem identified concerned the repetition of the level descriptors; in the English version, these are distinguished by slight changes in the adjective used in each sentence, e.g. ‘I have no problems in walking about; I have slight problems in walking about; I have moderate problems in walking about, etc’. In a written language, this format works because someone reading the questionnaire is able to scan between the level descriptors, which are all on the same page, make comparisons and reach a decision; there is simultaneous presentation of available choices. In a visual language, where the ‘text’ of the questionnaire is presented on screen via an online interface, repeating the level descriptors one after another is a sequential experience for the ‘viewer’. To compare the different options would require flicking between five different videos, separately presented on screen, which is not an equivalent cognitive task to the written version where there is simultaneous access to the range of responses from which to choose. After discussion with the forward translators and the representatives from the EuroQol group, a change in the format of presentation of the potential responses was permitted. The five-level descriptors are, for each domain, presented by a single signed phrase in the form of: ‘the health domain (e.g. mobility difficulties) followed by none; slight; moderate; severe; unable/can’t’. The grammar of BSL permits intensity to be marked in increasing degrees, having established the core subject first, through inflecting facial expression, handshape, movement and, in some instances, location of signing [16]. The viewer is able to see all possible choices of response simultaneously (as a reader of written text might) and come to their decision. Those taking the assessment give their response by clicking on one of the available choices represented on screen by corresponding English words (see Fig. 1 for a screen shot), and a BSL reference translation is given at the start and available to be seen again throughout if required.

#### Back translation

The two back translators independently translated the BSL version (second draft) back into English, compared the back translations with the original version and produced a report on the back translation process to the project manager. This resulted in the third draft.

#### Respondent testing

The third draft was tested using a sample of eight lay Deaf respondents (five men and three women), aged between 33 and 58, with varying educational backgrounds. They included both healthy people and patients, as outlined by the EuroQol group. The ‘patients’ were those who reported that they were currently experiencing health difficulties in response to general descriptive questions about their health and any current treatment. They were asked to complete the EQ-5D-5L BSL, not having had previous access to or experience of the assessment in English, and then they took part in a structured interview. Additionally, they completed a rating exercise (on a scale of 0–100) to establish their response for each health dimension, which confirmed that the severity descriptors for each dimension were appropriate. Feedback resulted in some additional changes to the translation, including clarification of the acronym EQ-5D, a clearer distinction between pain and/or discomfort and a greater emphasis on ‘today’ to be conveyed for each dimension as it was signed.

#### Testing with bilingual Deaf people

To explore agreement between the English and BSL versions of EQ-5D, 11 bilingual Deaf people completed both versions. Cohen’s kappa (*k*) statistic was used to assess the level of agreement between the English and BSL versions. Although the sample was small, it was found that the level of agreement between the two versions was very high. Statistical agreement (Cohen’s kappa) between the BSL and English versions of each dimension of EQ-5D-5L was high: Mobility, *k* = 1 (*p* < 0.001); Usual Activities, *k* = 1 (*p* < 0.001); Pain/Discomfort, *k* = 0.81 (*p* < 0.001); Anxiety/Depression, *k* = 1 (*p* < 0.001). For Self-Care, all respondents used only one category of the five levels for the English and BSL versions; thus, it was not possible to estimate Cohen’s kappa. The analyses above demonstrate that the content of each item in the BSL version of EQ-5D-5L was equivalent to the English items in the original version.

#### Fourth draft

This took into account comments from the respondent testing and the EuroQol translation review team and was the version then used for reliability testing. Examples of the amendments made include: (i) emphasising more strongly that the question is asking about the severity of problems for today only; (ii) making it clearer that the mobility domain refers to the ability to walk rather than barriers to mobility including communication barriers; and (iii) making the distinction between pain and discomfort clearer.

### Investigating the validity and reliability of the BSL version of EQ-5D-5L

Draft four of EQ-5D-5L BSL was uploaded to an adapted web platform, ‘Selectsurvey’, which allows videos to be embedded within it (https://selectsurvey.net/). This remote data capture technique is time-consuming and cost-effective and appropriate for a geographically dispersed, small linguistic community such as the Deaf community [13] whilst accommodating the visual modality of the language.

#### Sample size estimates

For the test–retest of EQ-5D-5L BSL (at baseline and one week later), an intra-class correlation coefficient (ICC) of at least 0.7 was required to establish reliability. Conventions used were ‘poor’ for ICC values less than 0.40, ‘fair’ for values between 0.40 and 0.59, ‘good’ for values between 0.60 and 0.74 and ‘excellent’ for values between 0.75 and 1.0 [19]. Typically, 0.7 is the minimum acceptable for research purposes [20]. A sample size of 51 allows a 95 % confidence interval for an ICC of 0.75 to be estimated to within plus or minus 0.1. The aim was to recruit 75 people in case of incomplete data. Previous studies demonstrated that this sample size, utilising the same method of recruitment, was entirely feasible [17, 21].

#### Recruitment

Participants were recruited using email, Facebook, word of mouth/hands and online message boards read or watched by Deaf people. For the purposes of assessing the reliability and validity of the BSL EQ-5D-5L within a participant sample, it was felt that the benefits of increased sample size from this recruitment approach outweighed the risks of selection bias. Inclusion criteria were: 18 years old or older and a Deaf BSL user. All information and consent materials were available in BSL, with an option for direct contact with a native BSL user for further clarification. Informed consent was obtained from participants online prior to completing the assessments, EQ-5D-5L BSL, CORE-10 BSL, CORE-6D BSL (see below) and this included consent to contact a participant’s GP (General Practitioner). If a participant gave an answer other than ‘never’ to the CORE-10 BSL question about suicidal intent, the research team regarded this as a flag for concern.

### Materials and procedure

Participants completed a short demographic survey, the EQ-5D-5L BSL and the CORE-10 BSL and CORE-6D BSL. Included in the demographic survey were questions relating to:

- Age and gender;
- Parental hearing status (an indicator of whether someone grew up with BSL as native language);
- A self-report of their current difficulties (if any) with their physical and/or mental health.

EQ-5D-5L was presented in a self-report on-screen format in BSL and accessed online. It has five levels of response (no problems, slight problems, moderate problems, severe problems and extreme problems) for each of the five dimensions of health. The standard EQ-VAS was also included. This asked the participants to rate their health on the day from 0 (‘the worst health state you can imagine’) to 100 (the best health state you can imagine’). The VAS was portrayed as an on-screen thermometer with a button that was moved to choose the placement upon it and then automatically captured the number relating to this position. In addition, there was a box for a participant to write in their choice of number from 0 to 100. Both approaches were used because the printed English version of EQ-5D-5L asks the participant to mark an X on a scale and to write the number from the scale into a box.

The CORE-10 [22] and the CORE-6D are self-report instruments designed to be used as screening tools for psychological distress; all items in both derive from the CORE-OM, which has previously been translated into BSL and its reliability demonstrated [21]. Two items in CORE-10 BSL and CORE-6D BSL are the same and were not duplicated. Therefore, a total of 14 items were presented on screen in their BSL form as previously established from the CORE-OM BSL. The CORE-10 BSL and the CORE-6D BSL have five levels of response: (0 = Never, 1 = Rarely, 2 = Sometimes, 3 = Often, 4 = Mostly/Always). The maximum possible score for the CORE-10 BSL is 40 and the lowest possible is 0; the maximum possible score for the CORE-6D BSL is 24 and the minimum 0.

### Analysis

The frequencies and percentages of responses on all items were calculated. A value of 1 on the EQ-5D-5L index score = ‘perfect health’ and 0 = ‘as bad as death’. The CORE-10 BSL responses were combined into a single unweighted score. This was calculated as the sum of the item scores divided by the number of questions answered. CORE-6D BSL is not a standalone measure. It was only used to report convergent validity with the EQ-5D-5L BSL.

Published population tariffs for the EQ-5D 3 level [23, 24] and the published crosswalk calculator (http://www.euroqol.org/about-eq-5d/valuation-of-eq-5d/eq-5d-5l-value-sets.html) were used to generate a preference weight for each possible combination of the EQ-5D-5L items and levels [25]. The index is calculated by deducting the appropriate weights from 1, giving a range of 1 or less. Negative values (reflecting health states considered to be worse than death) are possible [24]. The mean utility index score for the UK is 0.856 [24].

The published population norms for the UK were compared with results from the Deaf population sample. However, these are for the 3 level version of the EQ-5D [23], the study sample is relatively small, and the comparisons are not standardised for age and gender; therefore, only percentages with ‘no problems’ in each health domain are compared.

Cronbach’s alpha values were calculated to assess internal reliability of the translated items within EQ-5D-5L BSL, the five attributes of which are treated as different facets of the single construct of health-related well-being. One week after their first completion, participants were asked to complete the EQ-5D-5L BSL again to calculate reliability over time [by calculating the interclass correlation coefficient (ICC) using an absolute two-way mixed estimator]. Values above 0.75 are considered as an ‘excellent’ agreement between the first and second tests [26].

Weighted kappa scores were used to examine the reliability for the individual items of EQ-5D-5L BSL between the first and second tests: <0.20 (poor), 0.21–0.40 (fair), 0.41–0.60 (moderate), 0.61–0.80 (good), 0.81–1.00 (very good) [27].

The CORE-6D BSL and CORE-10 BSL measure aspects of health, as does the EQ-5D-5L; therefore, an overall relationship might be expected and a stronger relationship expected between specific items in each instrument measure. Five questions in CORE-10 BSL and one in the CORE 6D BSL cover the Anxiety/Depression domain; one question in CORE-6D covers Pain/Discomfort domain, and one question in CORE-6D covers the functioning domain of the EQ-5D-5L BSL. Kendall’s tau was used to assess the correlation between related items within each tool, which is a more robust estimator than Spearman’s rank correlation or Pearson’s correlation, especially on smaller sample sizes.

Discussion with the EuroQol representatives indicated they would expect an association between concurrent disability or health problems. Known-groups analysis, using the Mann–Whitney U test, was performed to confirm whether this existed or not. However, the sample size used in this study was not sufficient to assess whether participants’ demographic characteristics were statistically associated with the EQ-5D utility index.

## Results

### Interviews from the respondent testing stage

The interviews with eight Deaf people at the respondent testing stage indicated that how Deaf people understand health-related concepts is, in some cases, influenced by their experiences of communication. For example, a few people explained that, to them, ‘mobility’ encompassed the use of public transport (how easy would it be for a Deaf person to get around?). One respondent stated that he would select ‘slight problems’ because of the communication barriers rather than considering it from the perspective of physical ability. For others, when considering the Anxiety/Depression domain, they also were considering the linguistic accessibility of mental health services as an influence on their response, not just their internal distress.

### Psychometric properties of EQ-5D-5L BSL

One hundred people participated in the first test of EQ-5D-5L BSL (draft four version). Eight did not meet the inclusion criteria and were excluded from the data analysis; they either did not report their hearing status or were ‘hard of hearing’ (i.e. did not use BSL). Seventy-four of the original 92 Deaf people returned to take part in the retest. Of 100 people who originally completed the first survey, 18 responses to the suicidal intent question contained within CORE-10 (‘I have made plans to end my life’) triggered the study protocol to contact them and alert their primary care physician: 16 of these were included in the analysis; the remaining 2 did not meet the inclusion criteria.

Table 1 presents the demographic characteristics of the sample.

**Table 1:** Demographic profile of participants

	First test *n* = 92	Test–retest *n* = 74
Gender		
Female	64 (69.6 %)	52 (70.3 %)
Male	26 (28.3 %)	21 (28.4 %)
Missing data	2 (2.2 %)	1 (1.4 %)
Age		
18–24	4 (4.3 %)	4 (5.4 %)
25–34	14 (15.2 %)	10 (13.5 %)
35–44	17 (18.5 %)	14 (18.9 %)
45–54	26 (28.3 %)	22 (29.7 %)
55–64	14 (15.2 %)	11 (14.9 %)
65+	3 (3.3 %)	2 (2.7 %)
Missing data	14 (15.2 %)	11 (14.9 %)
Ethnicity		
Asian or Asian British: Indian	4 (4.3 %)	2 (2.7 %)
Asian or Asian British: Pakistani	3 (3.3 %)	1 (1.4 %)
Black or Black British: other Black background	1 (1.1 %)	1 (1.4 %)
Mixed: any other mixed background	1 (1.1 %)	1 (1.4 %)
Other ethnic group	3 (3.3 %)	1 (1.4 %)
White: any other white background	4 (4.3 %)	4 (5.4 %)
White: British	71 (77.2 %)	61 (82.4 %)
White: Irish	2 (2.2 %)	1 (1.4 %)
Missing data	3 (3.3 %)	2 (2.7 %)
Parents Deaf?		
Yes	24 (26.1 %)	21 (28.4 %)
No	68 (73.9 %)	53 (71.6 %)
Age first used BSL		
From birth	22 (23.9 %)	18 (24.3 %)
1–3 years old	20 (21.7 %)	15 (20.3 %)
4–7 years old	15 (16.3 %)	13 (17.6 %)
8–11 years old	6 (6.5 %)	6 (8.1 %)
12–16 years old	7 (7.6 %)	7 (9.5 %)
17–24 years old	13 (14.1 %)	7 (9.5 %)
25+ years old	9 (9.8 %)	8 (10.8 %)
Currently in employment		
Yes	63 (68.5 %)	52 (70.3 %)
No	28 (30.4 %)	21 (28.4 %)
Missing data	1 (1.1 %)	1 (1.4 %)
Health difficulties		
Yes	26 (28.3 %)	21 (28.4 %)
No	58 (63 %)	47 (63.5 %)
I don’t know	8 (8.7 %)	6 (8.1 %)

Nearly all had qualifications at GCSE level or above (95.3 %) and 41.2 % had at least an undergraduate degree or equivalent qualification. On a five-point scale, the majority of participants (78.3 %) identified with the two highest categories of considering themselves as ‘culturally Deaf’. A majority also reported that they are ‘often’ or ‘very much’ involved in the Deaf community (85.9 %) and have ‘a sense of community belonging’ (75 %). Nine per cent reported that they did not know whether they had any health difficulties or not. Table 2 presents the frequencies and percentages of responses on all items at the first test.

**Table 2:** Frequencies and percentages of responses for items within EQ-5D-5L BSL, CORE-10 BSL

Based on *n* = 92, *N* (%)					
EQ-5D-5L health states	None	Slight	Moderate	Severe	Extreme
EQ-5D-5L Mobility	63 (68.5)	18 (19.6)	6 (6.5)	5 (5.4)	0 (0)
EQ-5D-5L Self-Care	80 (86)	9 (9.7)	2 (2.2)	2 (2.2)	0 (0)
EQ-5D-5L Usual Activities	57 (61.3)	26 (28)	6 (6.5)	3 (3.2)	1 (1.1)
EQ-5D-5L Pain/Discomfort	45 (48.4)	31 (33.3)	7 (7.5)	6 (6.5)	4 (4.3)
EQ-5D-5L Anxiety/Depression	43 (46.2)	31 (33.3)	15 (16.1)	4 (4.3)	0 (0)

CORE-10 states	Never	Rarely	Sometimes	Often	Mostly/always
CORE-10 Q1	23 (26.4)	26 (29.9)	28 (32.2)	8 (9.2)	2 (2.3)
CORE-10 Q2	13 (14.9)	16 (18.4)	25 (28.7)	16 (18.4)	17 (19.5)
CORE-10 Q3	11 (12.8)	23 (26.7)	27 (31.4)	12 (14)	13 (15.1)
CORE-10 Q4	36 (40.9)	20 (22.7)	27 (30.7)	5 (5.7)	0 (0)
CORE-10 Q5	45 (51.1)	24 (27.3)	14 (15.9)	5 (5.7)	0 (0)
CORE-10 Q6	68 (79.1)	13 (15.1)	4 (4.7)	1 (1.2)	0 (0)
CORE-10 Q7	23 (26.7)	27 (31.4)	24 (27.9)	12 (14)	0 (0)
CORE-10 Q8	20 (23)	30 (34.5)	31 (35.6)	6 (6.9)	0 (0)
CORE-10 Q9	17 (19.5)	28 (32.2)	34 (39.1)	8 (9.2)	0 (0)
CORE-10 Q10	49 (57)	18 (20.9)	16 (18.6)	2 (2.3)	1 (1.2)

The distribution of the EQ-5D-5L BSL utility index was skewed and the median value was used to represent the average, which is 0.84 with 95 % CI [0.72–0.82 bias-corrected accelerated bootstrap (BCa)] (mean = 0.78, SD = 0.24, IQR = 0.72–1.00). The mean score for CORE-10 BSL is 11.74 (SD = 5.31) and was not skewed (for reference, the median = 11.50 with 95 % CI (10.64–13.12) BCa, IQR = 8.0–16.0).

**Fig. 2:**
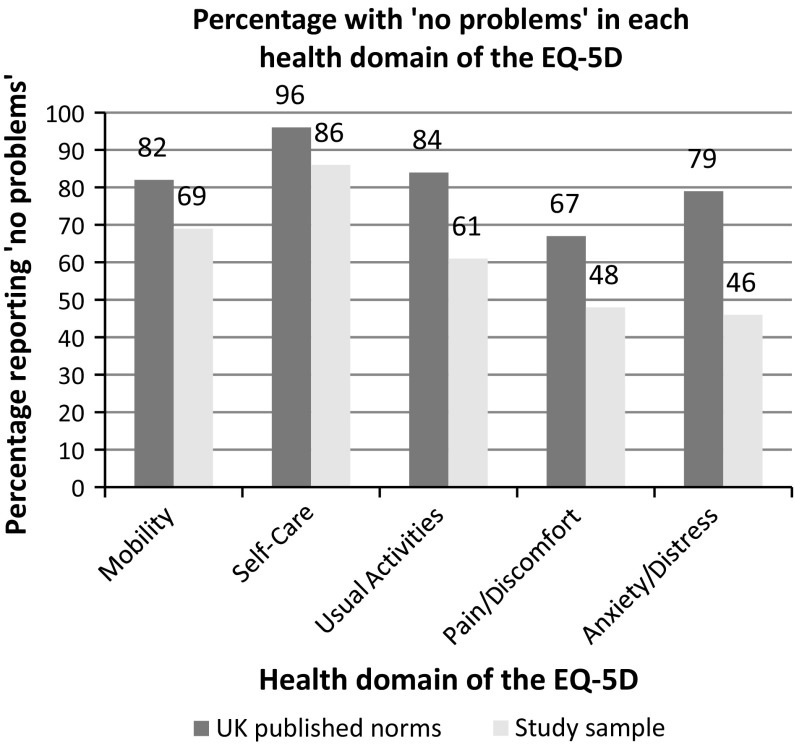
Bar chart showing the percentage with ‘no problems’ in each health domain of the EQ-5D

The mean utility index score for EQ-5D-5L BSL in this study was 0.78. The percentage of the study sample with ‘no problems’ in each health domain was less than the UK population published norms (Fig. 2).

The Cronbach’s alpha values for EQ-5D-5L BSL showed that the internal reliability is ‘good’ [*α* = 0.86 with 95 %CI (0.80–0.90), *n* = 89, for initial test; and *α* = 0.82 with 95 %CI (0.74–0.88), *n* = 72, for test–retest] and for CORE-10 BSL ‘acceptable’ [*α* = 0.72 with 95 %CI (0.62–0.80), *n* = 78, for initial test; and *α* = 0.75 with 95 %CI (0.65–0.83), *n* = 70, for test–retest] [28]. These values showed that there is good internal agreement between the items for each measure, but not so much agreement to suggest one or more items were redundant.

Participants were asked to repeat the measures one week later although some took longer. A sensitivity analysis on three subsets of the data showed that the values are consistent regardless of the time it took to retest: all data (*n* = 74) ICC = 0.87; two weeks or less to retest (*n* = 63) ICC = 0.87; seven days = /− three days to retest (*n* = 50) ICC = 0.86. The ICC for VAS and the typed score between two time points were also considered as excellent and good, respectively [ICC = 0.82 (*n* = 72) and ICC = 0.64 (*n* = 60), respectively].

The reliability for the individual items of EQ-5D-5L BSL between the first and second tests was examined using weighted kappa (see Table 3). The agreement was generally ‘moderate’ to ‘good’ [27].

**Table 3:** Weighted kappa values by question between first and second tests of EQ-5D-5L BSL

	Weighted kappa [95 % CI]
EQ-5D-5L BSL Mobility	0.61 (*n* = 73) [0.45, 0.77]
EQ-5D-5L BSL Self-Care	0.47 (*n* = 73) [0.08, 0.86]
EQ-5D-5L BSL Usual Activities	0.54 (*n* = 73) [0.38, 0.69]
EQ-5D-5L BSL Pain/Discomfort	0.61 (*n* = 73) [0.48, 0.74]
EQ-5D-5L BSL Anxiety/Depression	0.48 (*n* = 72) [0.32, 0.64]

The convergent validity for EQ-5D-5L BSL was assessed by checking how well it correlated with CORE-10 BSL and CORE-6D BSL at the first test (*n* = 92). The EQ-5D-5L BSL has a positive Pearson’s correlation with CORE-10 BSL and CORE-6D BSL (*r* = −0.432, *n* = 78, *p* < 0.001 and *r* = −0.449, *n* = 82, *p* < 0.001, respectively). Four of the five items from the CORE-10 BSL and two of the three items from the CORE-6D BSL demonstrated medium-strong correlation (≥0.3) [29] in the right direction with the EQ-5D-5L BSL Anxiety/Depression domain. One item from the CORE-10 BSL had a strong correlation (≥0.5) with EQ-5D-5L BSL Anxiety/Depression, and one item from the CORE-6D BSL demonstrated a strong correlation in the right direction with the Pain/Discomfort domain of the EQ-5D-5L BSL, but no significant correlation was found between one item from the CORE-6D BSL and the Usual Activities domain of the EQ-5D-5L BSL (Table 4).

**Table 4:** Convergent validity between items from the CORE-10 BSL and the CORE-6D BSL

CORE question	EQ-5D-5L domain	Kendall’s tau	Fisher’s exact test
CORE-10 Q1 (tense, anxious or nervous)	Anxiety/Depression	0.50	*p* < 0.001
CORE-10 Q5 (panic or terror)	Anxiety/Depression	0.379	*p* = 0.001
CORE-10 Q6 (end life)	Anxiety/Depression	0.295	*p* = 0.002
CORE-10 Q8 (despairing or hopeless)	Anxiety/Depression	0.410	*p* < 0.001
CORE-10 Q9 (unhappy)	Anxiety/Depression	0.319	*p* < 0.042
CORE-6D (I have felt terribly alone and isolated)	Anxiety/Depression	0.393	*p* < 0.001
CORE-6D (I have been troubled by aches, pains or other physical problems)	Pain/Discomfort	0.58	*p* < 0.001
CORE-6D (I have been able to do most things I needed to)	Usual Activities	−0.11	*p* = 0.366

The expected association between concurrent disability or health problems (yes/no) and utility weights estimated from the EQ-5D-5L BSL was confirmed (Mann–Whitney U, *p* < 0.001), with better (higher) values on the EQ-5D-5L BSL being associated with no problems.

## Discussion

The results demonstrate the psychometric properties of the EQ-5D-5L BSL are good, indicating that it can be used to measure health status and QALYs in the Deaf signing population in the UK. Convergent validity between EQ-5D-5L BSL and CORE-10 BSL and CORE-6D is consistent, demonstrating that the BSL version of EQ-5D-5L is a good measure of the health status of an individual. The test–retest reliability of EQ-5D-5L, for each dimension of health, was shown to have Cohen’s kappa values of 0.47–0.61; these were in the range of moderate to good and therefore acceptable.

Cohen’s kappa values in EQ-5D-5L BSL for Self-Care, Usual Activities and Anxiety/Depression were moderate, whereas for Mobility and Pain/Discomfort, they were good. The reasons for the moderate values for Self-Care and Usual Activities are not known. In relation to Anxiety/Depression, one possible explanation for the moderate agreement between the two tests is any emotional changes during the period of retest.

Nearly 9 % of Deaf participants in this study stated they *did notknow* if they had health difficulties, possibly indicating a lack of understanding of what is considered ‘being healthy’. This may result from poor access to health-related information generally as so little is available in BSL [6] or any signed language [4, 5]. The limited interview data from the respondent testing stage indicates that Deaf people’s responses to the health domains explored in the EQ-5D-5L may also be mediated by their experiences of communication barriers, with respect to both services and everyday life. Severity ratings of difficulties with mobility or anxiety may be influenced by experiences of whether services are accessible in BSL rather than only the severity of symptoms. Further study is required to explore Deaf people’s conceptualisation of ‘health’ and whether experiences of communication barriers mediate personal ratings of healthiness.

Cautious comparisons between study sample results and published EQ-5D UK population norms show a far lower percentage of Deaf people in this sample reported ‘no problems’ in the health domains of the EQ-5D in comparison with the general UK population. This is consistent with a recent study of Deaf health in the UK [6]. Further research is required to facilitate comparison of EQ-5D health states and utility values for the Deaf population in relation to the general population.

### Limitations

Participants may not be representative of the Deaf population, and collection online restricts the sample.

## Conclusion

This is the first signed version of the EQ-5D-5L instrument. It is a significant step forward in the study of cost-effectiveness and health status of Deaf people.
